# Persistent COVID-19 Infection in Wiskott-Aldrich Syndrome Cleared Following Therapeutic Vaccination: a Case Report

**DOI:** 10.1007/s10875-021-01158-5

**Published:** 2021-10-29

**Authors:** Rachel E. Bradley, Mark J. Ponsford, Martin J. Scurr, Andrew Godkin, Stephen Jolles, On behalf of the Immunodeficiency Centre for Wales, Kathryn Bramhall, Colin R. Price, Kimberly Evans, Emily Carne, Tariq El-Shanawany, Richard Cousins

**Affiliations:** 1 Immunodeficiency Centre for Wales, University Hospital of Wales, Cardiff, UK; 2 Division of Infection & Immunity, School of Medicine, Cardiff University, Cardiff, UK; 3 ImmunoServ LTD, Cardiff, UK; 4 Systems Immunity University Research Institute, School of Medicine, Cardiff University, Cardiff, UK; 5 Department of Gastroenterology & Hepatology, University Hospital of Wales, Cardiff, UK

To the Editor

Interventions to address viral persistence of COVID-19 in immunosuppressed individuals are urgently needed for both patient and societal benefit [1]. Infection with the novel pandemic coronavirus, SARS-CoV-2, has recently been reported up to 315 days in adults with hematological malignancies [2, 3]. These cases demonstrated recovery of infectious “live” virions and accumulation of mutations favoring vaccine escape during protracted SARS-CoV-2 infection [2, 3]. Immunodeficient individuals have thus been suggested as a reservoir for endemicity and a potential threat to the global vaccination effort. This mirrors wider experience with primary immunodeficiency, where excretion of neuro-virulent vaccine-derived polio virus has been described in association for up to 28 years [4]. To date, reports of protracted SARS-CoV-2 infections have focused on symptomatic cases, with varying therapeutic success [2, 3, 5]. The broad spectrum of SARS-CoV-2 presentations and frequently atypical nature of disease in immunosuppressed individuals led us to suspect viral persistence may also occur in pauci-symptomatic cases. Here we describe a case of protracted SARS-CoV-2 infection for 218 days in a patient with Wiskott-Aldrich syndrome (WAS), an inborn error of metabolism predisposing to dysregulated immunity and infection. We find that mRNA vaccination allowed induction of humoral and cellular immune responses to SARS-CoV-2, which had not been triggered by the ongoing infection itself, followed by viral clearance.

## Case Description

A 37-year-old Caucasian male with Wiskott-Aldrich syndrome (WAS) (IVS6 + 5G>A) developed anosmia and ageusia, followed by cough, dyspnoea, and fatigue (henceforth day 0). Community nasopharyngeal reverse-transcription PCR (RT-PCR) testing 1 day later was positive for SARS-CoV-2. Contact tracing suggested workplace exposure. Past medical history included childhood splenectomy for thrombocytopenia, eczema, recurrent infections, early bronchiectasis, asthma, and persistent molluscum contagiosum. Following diagnosis of specific antibody deficiency aged 24 years (based on suboptimal conjugated and unconjugated vaccine responses and reduced class switched memory B cells, described online supplementary), he was commenced on subcutaneous immunoglobulin replacement (SCIg) and maintained on 8 g/week with trough IgG levels of 12-15 g/L. Sinopulmonary infection history included RT-PCR-confirmed HKU1-coronavirus infection 12 months previously.

**Fig. 1:**
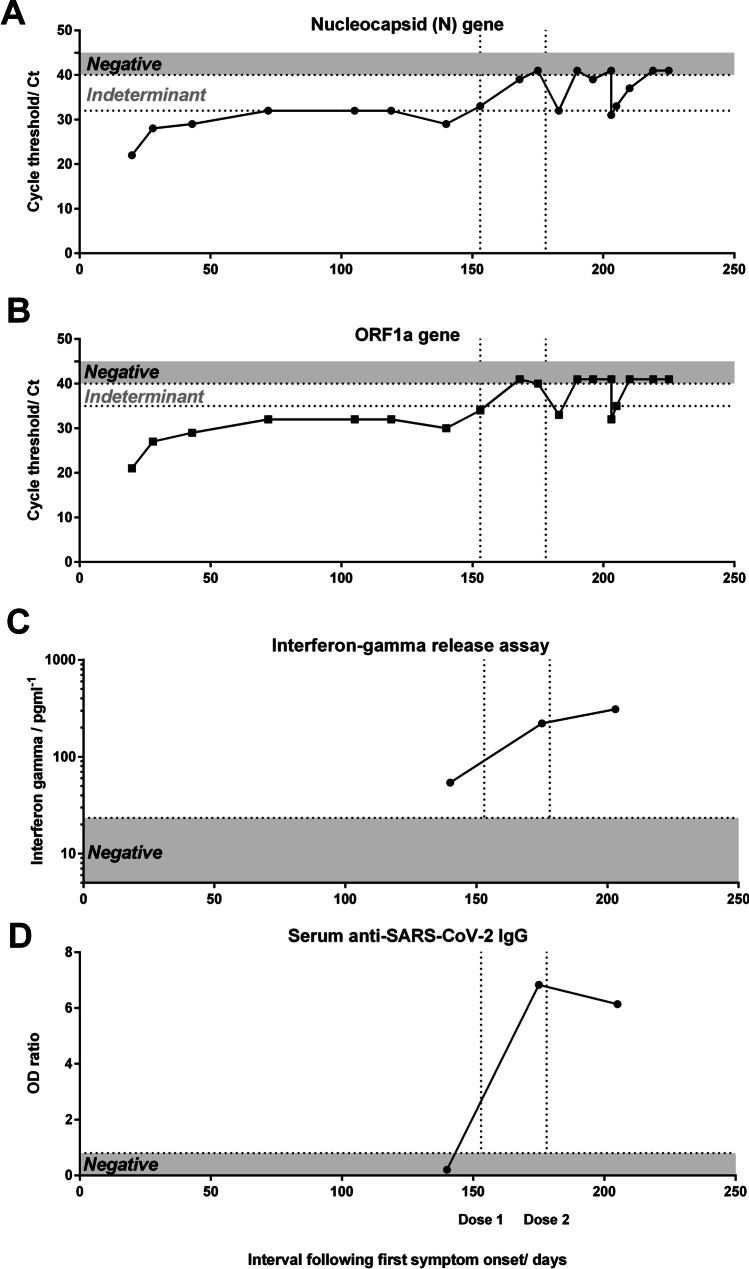
Viral load and immune response relative to vaccination. Semi-quantitative reverse transcriptase polymerase chain reaction (RT-PCR, Perkin-Elmer) detection of SARS-CoV-2 by **A** N-gene and **B** ORF1a-gene. **C**: interferon-gamma T cell responses to both SARS-CoV-2 nucleocapsid and spike peptide pool stimulation. **D** Anti-SARS-CoV-2 spike S1-domain IgG serum response. Vertical dashed lines indicate timing of 1st and 2nd doses of mRNA vaccination

The patient was monitored via our virtual COVID-19 ward. Home pulse oximetry showed maintained peripheral saturations >96% on room air and admission to hospital was not indicated. Incomplete improvement in sense of smell and taste was noted after 2 months; however, he experienced fluctuating symptoms of chest tightness, dyspnoea, poor concentration, and fatigue approximately every fourth week and serial RT-PCR testing remained persistently positive (Fig. 1A, B). Inflammatory markers such as C-reactive protein (CRP) remained less than 12 mg/L and computed-tomography (CT) imaging of the chest at day 153 showed small airway inflammatory changes with widespread bilateral basal tree-in-bud and centrilobular micro-nodularity, which had evolved from imaging 5 years prior (online supplementary, Figure S1). At day 140, anti-spike S1 SARS-CoV-2 IgG remained absent. Whole blood-based interferon-gamma (IFNγ) release assay showed an equivocal T cell response to a SARS-CoV-2 peptide megapool covering the spike, nucleocapsid, and membrane glycoproteins (see online supplementary), potentially reflecting prior HKU1-infection. The absence of serum neutralizing antibodies and immunocompromised status have previously been associated with isolation of infectious SARS-CoV-2 from the respiratory tract and in view of the individual’s occupation requiring close contact with members of the public, self-isolation was continued.

Given persistent RT-PCR positivity, ongoing fluctuating symptoms and impact on both work and mental health of prolonged self-isolation, we adopted an individualized therapeutic approach. A growing range of therapeutic strategies have been suggested to counter SARS-CoV-2 infection in immunocompromised individuals including antibody therapies (convalescent plasma (CPT), hyperimmune IgG, and monoclonal antibodies) and antivirals. Rapid symptomatic and virological response have been reported following convalescent plasma therapy (CPT) in B cell-deficient individuals with protracted SARS-CoV-2, including where antiviral therapy with remdesivir has failed [5]. However, use of CPT has been questioned following suggestion it may accelerate vaccine-escape mutations [3]. Passive immunization with neutralizing monoclonal antibodies appear efficacious in post-exposure settings, particularly in seronegative individuals; however, this was not clinically accessible [1]. We hypothesized stimulation of the endogenous immune response through therapeutic vaccination could support viral clearance. Two doses of bnt162b2 mRNA vaccination were administered 1 month apart, with mild flu-like symptoms only. Enhanced cellular responses and seroconversion were demonstrated 14 days following the first vaccine dose (Fig. 1C, D), with anti-SARS-CoV-2 spike IgG response exceeding thresholds commonly used for selection of convalescent plasma therapy. SARS-CoV-2-specific IFN-γ^+^ T cell increased significantly following vaccination to levels comparable to those induced among healthy controls. Anti-SARS-CoV-2 antibodies in immunoglobulin SCIg replacement were negative at this time (data not shown). A contemporaneous rise in RT-PCR cycle threshold (Ct) for detection by 6 cycles was noted across both viral nucleocapsid (N)-gene and ORF1a-gene targets. Given a difference of 1 Ct unit is approximately equivalent to a factor of 2 in the number of viral particles per sample, this represents a 64-fold decrease in viral genetic material recovered by nasopharyngeal swabbing 2 weeks following initial vaccination. SARS-CoV-2 clearance followed 72 days following first therapeutic vaccination dose (day 218 following initial RT-PCR detection).

## Discussion

This case highlights persistent SARS-CoV-2 infection as an important outcome in immunodeficient individuals, beyond commonly used metrics of mortality or critical illness. Persistent infection is often associated with a symptomatic burden to the individual, further amplified here by the psychological and economic impacts of prolonged isolation. Diagnosis may be reliant on a high index of clinical suspicion in order to minimize the risk of cross-infection, particularly in the hospital setting. To our knowledge, this is the first description of therapeutic mRNA vaccination in the context of persistent SARS-CoV-2 infection. Importantly, vaccination proved well-tolerated and successfully elicited humoral and cellular responses which had not been induced after 120 days of PCR-confirmed SARS-CoV-2 infection. The timing of viral clearance following induction of host immunity strongly supports a causal role for vaccination; however, we cannot exclude that viral clearance may have occurred independently. Further studies are required to assess the reproducibility and generalizability of our findings, particularly given the likely heterogeneity of vaccine-induced immune response across the spectrum of inborn errors of immunity. In conclusion, we highlight the potential for therapeutic vaccination in persistent SARS-CoV-2 infection where sufficient immunological function remains to produce relevant humoral and T cell responses.

## Supplementary Information

Below is the link to the electronic supplementary material.

Supplementary file1 (DOCX 185 KB)
