# Supplementary material for: Persistent COVID-19 Infection in Wiskott-Aldrich Syndrome Cleared Following Therapeutic Vaccination: a Case Report
Source: J Clin Immunol. 2021 Oct 29;42(1):32–5. doi: 10.1007/s10875-021-01158-5 (PMC8554737; doi:10.1007/s10875-021-01158-5)
Supplement: Supplementary file 1 — Supplementary file1 (DOCX 185 KB) [file 10875_2021_1158_MOESM1_ESM.docx]

***Online supplementary***

*Extended case description*

Wiskott Aldrich Syndrome (WAS) was diagnosed at birth based on thrombocytopenia, eczema and family history with genetic confirmation c.449+5G>A mutation (IVS6+5G>A), previously reported to result in incomplete normal splicing and generation of a cryptic splice site. Childhood wheeze and eczema accompanied recurrent skin and sinopulmonary infections despite prophylactic and rescue antibiotics. Splenectomy at 6 years was followed by improvement in thrombocytopenia. A diagnosis of specific antibody deficiency was made based on failure to respond to vaccination with Menitorix (protein conjugate, Hib/Men C) and pneumovax II (polysaccharide). Pre/post-vaccination responses were <0.11 ug/ml to 0.13 ug/ml; and 29.00 to 34.00 U/ml, respectively. Reduced class-switched memory B-cells 2.69% (interquartile range: 9.2-18.9) and low naïve CD8 T cells at 10x10^6^ cells/L (63-313x10^6^/L) were also noted.

*Extended methods*

T cell responses to SARS-CoV-2 were measured using a commercially available whole blood assay (ImmunoServ Ltd), as described elsewhere (1). Briefly, 10ml venous blood samples were collected into sodium heparin vacutainers (BD) and stimulated with peptides spanning the entire spike (S1 and S2) protein, nucleocapsid phosphoprotein and membrane glycoprotein. Samples were incubated at 37°C for 20-24 hours, before harvesting the plasma to analyse for IFN-γ by ELISA. A positive SARS-CoV-2-specific T cell response was defined as >23.55pg/ml IFNg and 50% above the negative (unstimulated) control value, as previously determined in healthy donors. Semi-quantitative reverse transcriptase polymerase chain reaction (RT-PCR) and anti- SARS-CoV-2 spike S1 IgG serology were determined using the Perkin Elmer and EuroImmun assays, respectively, according to manufacturer protocols. IgG antibody titres against Tetanus, Haemophilus influenzae, and streptococcus pneumoniae capsular polysaccharide were assessed by ELISA (The Binding Site, Birmingham, UK).

References

1. Scurr MJ, Zelek WM, Lippiatt G, Somerville M, Burnell SEA, Capitani L, et al. Whole blood-based measurement of SARS-CoV-2-specific T cell responses reveals asymptomatic infection and vaccine efficacy in healthy subjects and patients with solid organ cancers. medRxiv. 2021 Jan 1;2021.06.02.21258218.

Supplementary Figure 1: Absence of typical COVID-19 pneumonitis on pulmonary imaging


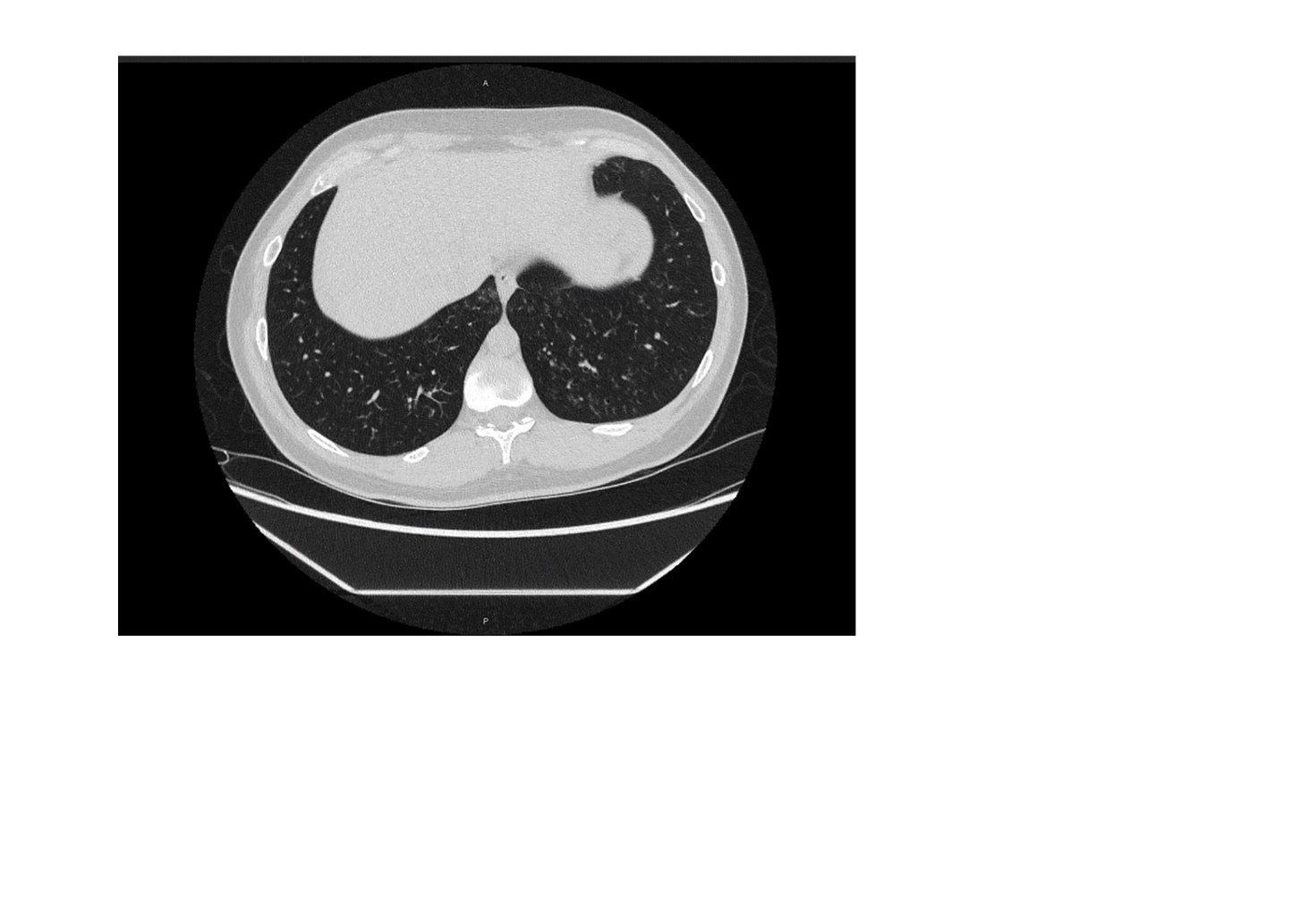


Computed-tomography (CT) chest imaging conducted on day 153 following onset of symptoms. Widespread bilateral basal tree-in-bud and centrilobular micro-nodularity (evolved from imaging 5 years prior) were present, without typical features of COVID-19 pneumonitis.
